# Guidelines to electrode positioning for human and animal electrical impedance myography research

**DOI:** 10.1038/srep32615

**Published:** 2016-09-02

**Authors:** Benjamin Sanchez, Adam Pacheck, Seward B. Rutkove

**Affiliations:** 1 Department of Neurology, Beth Israel Deaconess Medical Center, Harvard Medical School, Boston, MA 02215-5491, USA

## Abstract

The positioning of electrodes in electrical impedance myography (EIM) is critical for accurately assessing disease progression and effectiveness of treatment. In human and animal trials for neuromuscular disorders, inconsistent electrode positioning adds errors to the muscle impedance. Despite its importance, how the reproducibility of resistance and reactance, the two parameters that define EIM, are affected by changes in electrode positioning remains unknown. In this paper, we present a novel approach founded on biophysical principles to study the reproducibility of resistance and reactance to electrode misplacements. The analytical framework presented allows the user to quantify a priori the effect on the muscle resistance and reactance using only one parameter: the uncertainty placing the electrodes. We also provide quantitative data on the precision needed to position the electrodes and the minimum muscle length needed to achieve a pre-specified EIM reproducibility. The results reported here are confirmed with finite element model simulations and measurements on five healthy subjects. Ultimately, our data can serve as normative values to enhance the reliability of EIM as a biomarker and facilitate comparability of future human and animal studies.

For any electrophysiological measure to be effective as a diagnostic tool it needs to be highly reproducible. Yet the reproducibility of such measures is very dependent on the consistency of electrode placement. One example is the compound muscle action potential (CMAP), which is known to be sensitive to small movements of electrode position of either the active (E1) electrode, placed over the midpoint of muscle, or the reference (E2) electrode placed over the joint or tendon^1^,^2^,^3^. Changing the position of the active and reference electrodes will alter the shape and amplitude of the waveform recorded^4^,^5^, and this effect will be different depending on the muscle^6^,^7^. For example, a study showed in the extensor digitorum brevis muscle, the area where the CMAP amplitudes were at least 80% of the maximum CMAP amplitude represented a region of susceptibility (only 1.7 cm^2^), i.e. moving the electrode 7 mm away from the maximum site would cause an amplitude drop of more than 20%^6^. The flexor digitorum brevis muscle was found, however, less sensitive to changes in electrode positions, with region of susceptibility of 18.4 cm^2 ^6. Thus, care must be applied placing the electrodes to record the maximal CMAP amplitude^2^,^4^.

Electrical impedance myography (EIM)^8^ is a biomarker that can detect the morphological and pathological changes that accompany the onset and progression of neuromuscular diseases affecting muscle. Like CMAP, EIM’s reliability depends on the precise positioning of the electrodes. The working principle of EIM consists of applying a non-ionizing, alternating electrical current signal across the muscle of interest using two outer surface electrodes and then measuring the resultant voltage signal with two additional inner electrodes. The amplitude ratio and phase lag between the current and voltage signals defines the so-called “electrical impedance” at that particular frequency (typically in the kHz-MHz range)^9^,^10^,^11^,^12^. The practical relevance of EIM as a non-invasive and painless biomarker has been demonstrated extensively in the recent years in a variety of disorders, including spinal muscular atrophy^13^,^14^, amyotrophic lateral sclerosis^15^,^16^, and Duchenne muscular dystrophy^17^,^18^.

Guidelines for the clinical utilization of EIM require the four electrodes to be aligned over a specific area of the muscle of interest and in contact with the skin. This procedure can be particularly important during the course of longitudinal studies, where it is challenging to place all four electrodes at identical electrode sites with consistent distance between electrodes. Figure 1 illustrates the consequence of a poor positioning of the electrodes. In the figure, we show two extreme cases where the resistance *R* and reactance *X*, the two parameters that define the impedance, are measured after moving the high current source electrode ±75% the uniform distance between electrodes along the rectus femoris on a healthy subject. The reader can see the effect of electrode positioning in the impedance: the resistance and reactance increase or decrease depending on the electrode’s position, and the sensitivity is different when the current source electrode moves distally or proximally.

By providing a detailed analysis on the influence of electrode positioning in muscle impedance, we hope to improve EIM’s reliability as a biomarker for human and animal research as well as the comparability of future studies. This paper makes three major contributions. Firstly, we present a novel analytical framework to study the effect of non-aligned or non-evenly spaced electrodes on muscle impedance. Secondly, we report quantitative estimates on the relationship between the placement of the electrodes and the intra-class correlation. Lastly, we provide normative values of the precision needed to position the electrodes and the minimum muscle length needed for a pre-specified EIM reproducibility.

## Review of Previous Work

The vast majority of studies available evaluating the influence of electrode positioning in electrical impedance have been published in whole body bioelectrical impedance analysis (BIA). In wrist-to-ankle BIA measurements^19^, the standard placement of the current electrodes is the right hand and foot on the dorsal surfaces, proximal to the metacarpal-phalangeal and metatarsal-phalangeal joints. The potential electrodes are placed few cm apart from the current electrodes, with the center on the mid-line between the prominent ends of the right radius and ulna of the wrist, and mid-line between the medial and lateral malleoli of the right ankle. In these studies, the main objective has been to quantify the effect of a misplacement in one or more electrodes in the determination of body composition parameters. For example, it has been reported that 1 cm and 4 cm differences between electrode placement provokes changes in whole body resistance by 2%^20^,^21^ and 4.4%^20^, respectively. Another study, however, has reported a 1% change in fat mass when the electrodes were misplaced by 1 cm^22^. Of interest, most of these efforts have investigated only the effect on the resistance^20^,^23^,^24^,^25^, because the models used for assessing body composition parameters do not consider the reactance^26^. Importantly, most previous BIA studies have relied on statistics^19^, rather than biophysical or measurement^27^ principles, to understand the cause and effect of electrodes’ misplacement in the measured impedance.

Unlike BIA where the impedance is used for metabolic purposes, in EIM the interest is to detect alterations in muscle accompanying neuromuscular disorders by changes in resistance and reactance. We only know of two studies evaluating the sensitivity of EIM to the positioning of the electrodes. In the first study, the authors presented the robustness of the phase angle (obtained dividing the reactance by the resistance) at 50 kHz in front of electrode misplacements. In doing so, the authors placed the current electrodes on the dorsum of both hands for measuring the biceps and on the dorsum of bother feet for the tibialis anterior^28^. This electrode configuration, however, is no longer employed due to its relative inconvenience of application and the fact that alterations in muscle girth and limb position can alter the outcomes. In the second study, the results were limited to the measurement of the right quadriceps of a single individual^29^. Like^28^, one of the electrode topologies considered had the current electrodes placed on the dorsum of bother feet. Also, the author studied only the effect of a constant misplacement of the voltage electrodes in one direction. In both EIM studies, the results were not normalized to the distance between electrodes and so the values reported are only valid to the electrode separation used by the authors.

A review of the literature shows that a detailed analysis of the influence of electrode positioning in muscle impedance is lacking or incomplete (Table 1). We believe ours is the first study to consider all possible single and double electrode misplacements and their effects on EIM data, and confirm the results with biophysical theory and modeling. In the analysis that follows, the four electrodes were placed over the muscle of interest in accordance with the current standard configuration for measuring EIM. Moreover, we consider the most realistic and general case whereby the electrode positions are independent of each other in both directions. Finally, our results are normalized so as to allow prediction of precision needed for positioning the electrodes in both human and animal studies to achieve a pre-specified reproducibility.

## Results

### Theoretical results

We propose the following model to analyze the effect of electrode positioning in EIM,


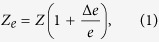


where *Z*_*e*_ = *R*_*e*_ + *jX*_*e*_ is the muscle impedance measured with electrodes’ misplacements, *Z* = *R* + *jX* is the “error-free” muscle impedance measured with uniform electrode spacing and *j* is the complex imaginary unit. The complex term Δ*e*/*e* exemplifies the idea of relative errors in the muscle impedance *Z*_*e*_ caused by errors positioning the electrodes. We based our model on similar concepts used in the fields of signal processing and statistics, considering the observation of a signal disturbed by additive noise. Using the properties of complex numbers, it is possible to find from (1) the expression for the error term Δ*e*/*e* that depends explicitly on the muscle resistance and reactance, namely


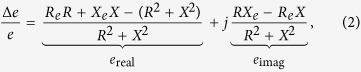


where *e*_real_ and *e*_imag_ are, respectively, the real and imaginary relative error terms affecting the muscle resistance and reactance. The relationship between Δ*e*/*e* and the relative resistance and reactance errors Δ*R*/*R* and Δ*X*/*X* can be found easily by combining equations 1 and 2,


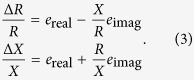


Importantly, using (3) to calculate Δ*R*/*R* and Δ*X*/*X* requires *a priori* knowledge on the muscle reactance and resistance values – in other words this approach can only be used in practice after acquiring experimental data. As a consequence, Δ*R*/*R* and Δ*X*/*X* will contain additional measurement errors beyond those solely affected by the positioning of the electrodes^30^,^31^, which can lead to a misinterpretation of the results.

To understand how Δ*R*/*R* and Δ*X*/*X* depend exclusively on the positioning of the electrodes, we present an analytical method that allows the quantification of EIM additional errors caused by the misplacement of electrodes. For the sake of simplicity, we analyze the influence of electrode positioning in longitudinal and transverse directions separately. First, we determine the muscle impedance measured with uniform electrode spacing *d* in equation 1^32^


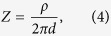


where *ρ* is the complex resistivity of the muscle^33^ (Supplementary information). Then, Table 2 reports the equations to calculate Δ*e*/*e* considering single and double electrode changes in position shown in Supplementary Figure 1.

Several important outcomes emerge from Table 2. The first major result is Δ*e*/*e* compiled in Table 2 have imaginary parts that are zero. Forcing *e*_imag_ = 0 in (2), we find *X*/*R* = *X*_*e*_/*R*_*e*_ and that is a simple and elegant proof that the phase is unaffected by the lack of precision positioning the electrodes. Then forcing *e*_imag_ = 0 in (3), we find the relative resistance and reactance experimental error is the same, i.e. Δ*e*/*e* = Δ*R*/*R* = Δ*X*/*X*. The second major result follows from observing that the errors in Table 2 do not depend on the electrical properties of the muscle or frequency measured: Δ*R*/*R* and Δ*X*/*X* depend only on the ratio between the electrode misplacement and the uniform electrode spacing. This observation will become relevant when the framework is validated with multi-frequency experimental data.

Finally, it is worth noting that the equations in Table 2 are analogous to the calculation of *e*_real_ with experimental data in (2). The great advantage of having Table 2 is that now we can predict the consequence of electrode(s) being misplaced in the resistance and reactance data without requiring experimental data. For example, a misplacement of the current injecting electrode of 5% along the major direction of the electrode array results in a 4% error in the muscle resistance and reactance. This result is valid for both healthy and diseased muscle, and it is independent of the frequency measured.

### Experimental results

**Figure 2:**
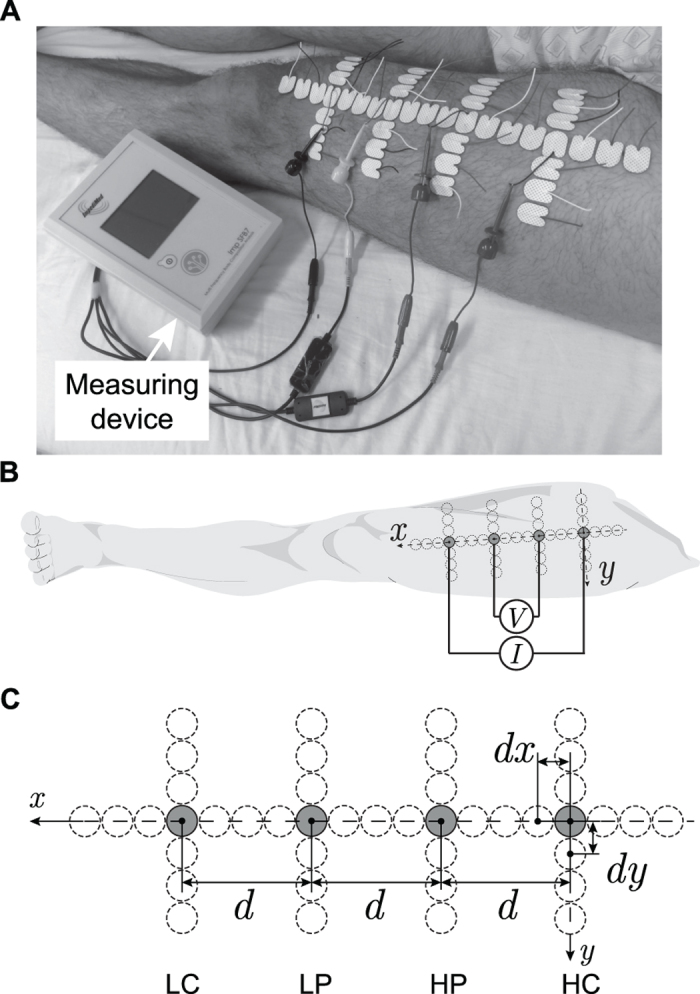
Electrode array configuration for evaluating the influence of electrode misplacements. Example of the experimental setup (**A**). Schematic representation showing the connection of the current (*I*) and voltage (*V*) electrodes (2 × 1.5 cm) to the measuring device (**B**). Detail of the electrodes arrangement (**C**). The dotted circumferences represent the different electrodes positions measured in *x* (longitudinal) and *y* (transverse) axes. The convention adopted is that a positive electrode misplacement in the *x* or *y* axis moves that electrode distally or laterally, respectively. The electrode configuration with a uniform spacing is *d* = 7 cm and is denoted with the electrodes in gray color. The neighboring electrode sites had a distance between centers with respect to adjacent electrodes *dx* = *dy* = 1.5 cm, giving *dx*/*d* and *dy*/*d* up to ±75% with increments of 25%. Electrodes nomenclature: HC, high current; HP, high potential; LP, low potential; LC, low current.

Below, we purposely avoid referencing the position of the electrodes by their anatomic orientation terms. Instead, we use the convention defined in Fig. 2 by the *x*, *y* axes so that the interpretation of the results is the same with the electrodes swapped position.

#### Effect of single electrode misplacement on muscle impedance

**Figure 3:**
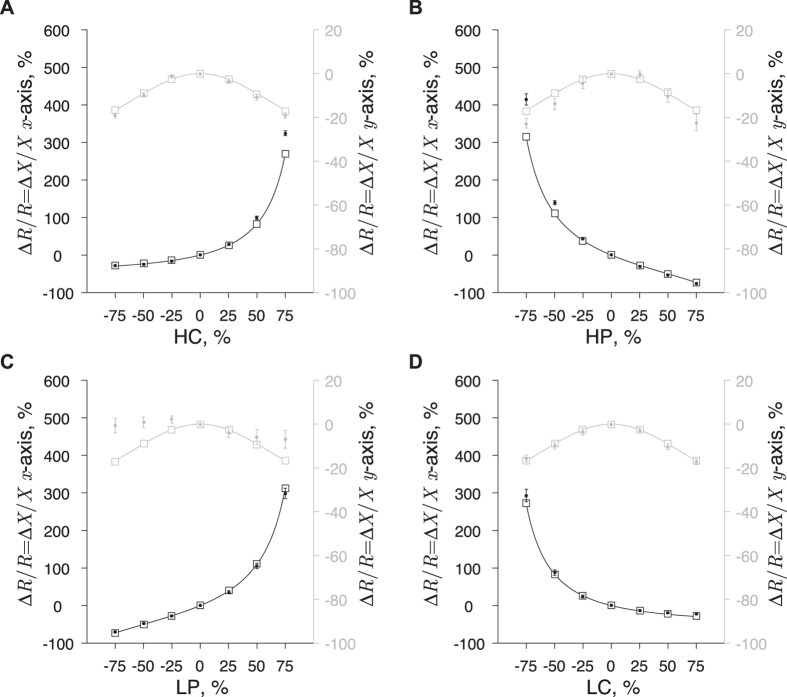
Influence of single electrode misplacement in the relative resistance and reactance errors: HC, high current (**A**); HP, high potential **(B**); LP, low potential (**C**); and LC, low current (**D**). In solid lines, the predicted resistance and reactance relative errors from Table 2. The resistance and reactance relative errors obtained with the three-dimensional finite element model are shown in white squares. The circles and horizontal error bars are the mean and standard error of the mean of the resistance and reactance relative errors measured at 50 kHz. Note that in many of the electrodes’ misplacements considered, the mean symbols obscure the error bars. Data in the *x* and *y* axes are shown in black and gray, respectively. We refer the reader to Table 3 for the accuracy comparison between the predicted and measured relative errors.

Figure 3 shows the muscle resistance and reactance values decreases if one electrode, regardless of which, is moved in the *y*-axis. The effect on the resistance and reactance values in the *y*-axis is the same for all the electrodes and it is found to be symmetrical around 0%; a result that is not unexpected given the electrodes’ symmetry with respect to the *x*-axis. In the *x*-axis, the resistance and reactance values increase exponentially when the current electrodes approach the voltage electrodes and *vice versa*. In contrast, the resistance and reactance decrease and reach a plateau at −29% when the current electrodes move farther from the potential electrodes, or they decrease further than −73% when the potential electrodes approach to each other beyond 75%. Interestingly, the (absolute) effect caused by an electrode misplacement in the *y*-axis is lower than that in the *x*-axis for all deviations from even spacing. This result emphasizes the relevance of a correct positioning of the electrodes, particularly in the main direction specified by the electrode array where the influence of an electrode positioning error on the resistance and reactance is greater.

The reader can see Fig. 3 the finite element model (FEM) simulations match very well the effect predicted by the analytical framework. Simply put, the FEM simulator solves numerically the same equations we solved analytically. The accuracy between the measured resistance and reactance relative error at 50 kHz and the predicted values is quantified in Table 3. In our framework, we assumed the electrodes moved on the *xy* plane (*z* = 0) on top of the muscle. While this can be considered a good approximation along the rectus femoris, there is a change in the *z*-axis due to the muscle girth when considering a lateral or medial electrode misplacement. Therefore, data with ±75% positioning error (a lateral and medial electrode misplacement of ±5.25 cm) were not included in Table 3 because they did not fulfill the previous assumption. Overall, the experimental results agreed satisfactorily with those of theory within 10% discrepancy.

The results above show the effect of electrode misplacement on the muscle resistance and reactance data does not depend on the frequency measured. To verify this experimentally, we calculated the resistance and reactance relative errors at only 133 frequencies between 10 and 200 kHz. We analyzed a limited number of frequencies measured at low frequencies to prevent our interpretation from being affected by high frequency errors introduced by cable capacitance and capacitance between electrode leads^30^,^31^. The difference between multi-frequency and single-frequency at 50 kHz data reported in Table 3 was proved to be not statistically significant when considering {−50, −25, 25, 50}% electrode positioning errors (*p* ≥ 0.55 in both directions).

#### Effect of double electrode misplacements on muscle impedance

**Figure 4:**
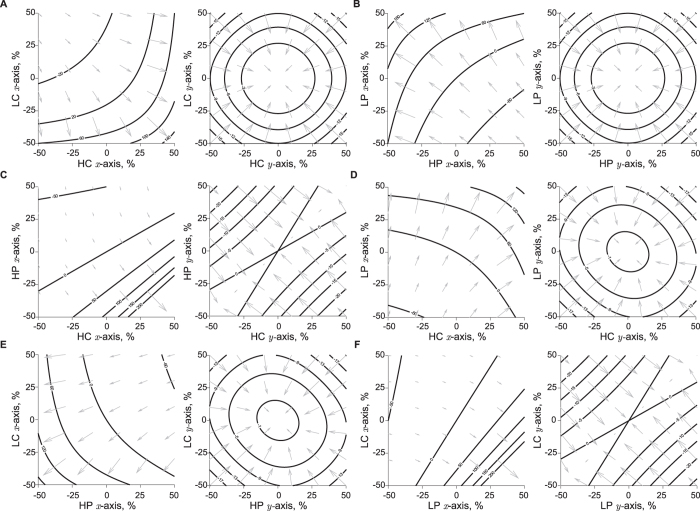
Influence of double electrode misplacements in the relative re resistance and reactance errors: HC & LC, high current and low current (**A**); HP & LP, high potential and low potential (**B**); HC & HP, high current and high potential (**C**); HC & LP, high current and low potential electrodes (**D**); HP & LC, high potential and low current (**E**); and LP & LC, low potential and low current (**F**). In solid lines, the predicted resistance and reactance relative errors from Table 2. The arrows in gray indicate the directions of the increasing resistance and reactance relative errors.

Representing and quantifying the effect of double electrode misplacements in the resistance and reactance values is more challenging than single electrode misplacement described above. In this case, the positioning error of each electrode is independent from each other and thus can only be plotted in a three-dimensional coordinate system. To help the reader to grasp the details, we show in Fig. 4 how the resistance and reactance are affected in a contour plot, where the isoline relative errors can be interpreted as if they were lines of same height in a topographic map. The arrows in gray are shown to indicate the directions of the increasing effect. The maximum (absolute) percentage difference between the values predicted and that found from FEM modeling was 0.8%.

#### The precision in positioning the electrodes affects data reliability

The analytical framework presented also allows the user to obtain quantitative estimates of the precision needed positioning the electrodes to achieve a pre-specified reproducibility. To generate muscle impedance values (resistance or reactance), we created in MATLAB the patient data set “measured” by the first examiner containing 100 synthetic values linearly distributed between 100 Ω and 250 Ω. The second patient data set, which contained other 100 synthetic values, was found from the first data set by adding 100 single electrode misplacements using the equations in Table 2. We considered changes in position of the electrodes as a Gaussian random variable to mimic the random effect in electrode placement. The reader can interpret the second data set as if there were a second examiner that repeated the procedure and misplaced the electrode position. Intuitively, one can see the measurements become less reproducible as the misplacement of the electrode increases. This observation leads to two important questions. First, how does the EIM reproducibility depend on the uncertainty in placing the electrodes? And second, does the EIM reproducibility depend in the same way for all the electrodes in both directions?

**Figure 5:**
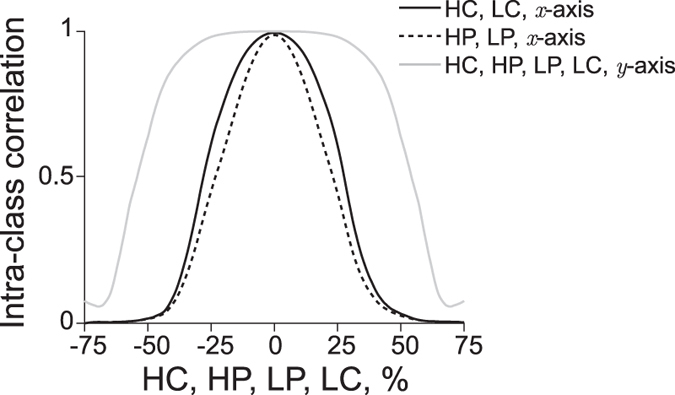
Evolution of intra-class correlation coefficients (ICC) from electrical impedance myography (EIM) test-retest data. The consequence of an increasing uncertainty positioning the electrodes is a decrease in EIM’s ICC values. ICC values in the *x*-axis: high current, solid gray; high potential, dotted black; low potential, dashed black; low current, dashdot black. In the *y*-axis, the ICC values are the same for all the electrodes, shown in dashed gray. We refer the reader to Table 4 for the numerical values. Electrodes nomenclature: HC, high current; HP, high potential; LP, low potential; LC, low current.

Assuming no other errors present during the measurement, if the second examiner places the electrodes at the same exact location as the first examiner, the electrode misplacement is zero, both impedance data sets are identical, and the intra-class correlation coefficient (ICC) is 1. As discussed above, the ICC coefficient decreases as the errors positioning the electrodes increases. Interestingly, the loss of reproducibility as the misplacement of the electrode increases has a strong symmetry about 0. In other words, the loss of reproducibility does not have a preference in the direction of the electrode misplacement (Fig. 5). It also shows the ICC coefficient decreases dramatically when the misplacement in the *x*-axis affects the high and low potential electrodes and that dependence is practically the same between the two potential electrodes. As for the high and low current electrodes in the *x*-axis, the loss of reproducibility is less severe than the potential electrodes. In the *y*-axis, the loss of reproducibility is the same for all four electrodes. Moreover, the reproducibility is higher in the *y*-axis because the effect of an electrode misplacement in the *y*-axis is less than the *x*-axis (Fig. 3). Table 4 provides the quantitative values for the maximum permissible uncertainty in the positioning of the electrodes in order to achieve different levels of reproducibility. Note that when EIM is affected by the errors positioning the electrodes, EIM’s reproducibility does not improve changing the frequency measured.

The results in Table 4 allow one to know the precision needed positioning the electrodes to improve EIM’s reliability. For example, in order to achieve an ICC value ≥0.8, the high current electrode must be placed along the *x*-axis in the muscle with an uncertainty ≤18.5%. That means that if we consider using an electrode array geometry with a uniform electrode spacing *d* = 1 cm, the maximum permissible electrode misplacement becomes 1.8 mm. However, if the uniform electrode distance increases to *d* = 5 cm, then the maximum permissible electrode misplacement increases to 9 mm. Indeed, replacing a gel-adhesive electrode with an uncertainty less than 9 mm is easier to achieve than 1.8 mm. This result proves that if the distance between electrodes is greater, then a high reproducibility in test-retest experiments and longitudinal studies will be easier to obtain. However, increasing the electrode distance comes at a cost: the penetration depth of the current that determines the portion of muscle that can be measured increases with the separation of the current electrodes^34^. As a consequence, the impedance measured may be affected by the presence blood volume in the vessels and arteries. Also, for some muscles, achieving the adequate distance for a specified reproducibility may be difficult or anatomically impossible. The logical question to ask is: what is the minimum distance *d*_min_ required between electrodes to ensure a pre-specified EIM reproducibility?

To answer this question, empirical assessment of the maximum uncertainty placing each of the electrodes multiple times is necessary or, otherwise, one must assume a reasonable value. Then, the user can divide the maximum uncertainty value by the minimum permissible misplacement of the electrodes using the values compiled in Table 4. In the example above, assuming that the examiner cannot place the high current electrode with an uncertainty below 1 cm in the *x*-axis along the muscle, and the ICC desired is 0.8 or greater, the minimum distance between electrodes is *d*_min_ = 1/0.18 = 5.4 cm. Equivalently, this is a minimum muscle length of 16.2 cm (the minimum length of muscle that can be measured is at least 3*d*_min_, the total length of the electrode array shown in Supplementary Figure 2A). Considering the same reproducibility, if the uncertainty placing the electrodes also affects the positioning of any potential electrode, then the distance becomes *d*_min_ = 1/0.13 = 7.7 cm, i.e. a minimum muscle length of 23.1 cm. Note that by only changing the electrodes being moved, the minimum muscle length increases 6.9 cm. If a better reproducibility is desired, e.g. ICC ≥ 0.9, then the minimum distance between electrodes increases *d*_min_ = 1/0.087 = 11.5 cm, and consequently the minimum muscle length increases to 34.5 cm. Also, the reader should note that because the effect of electrode misplacement is normalized to the distance between the electrodes, the values in Table 4 can be used to determine *any* muscle length measured with a uniform electrode array, whether animal or human.

**Figure 6:**
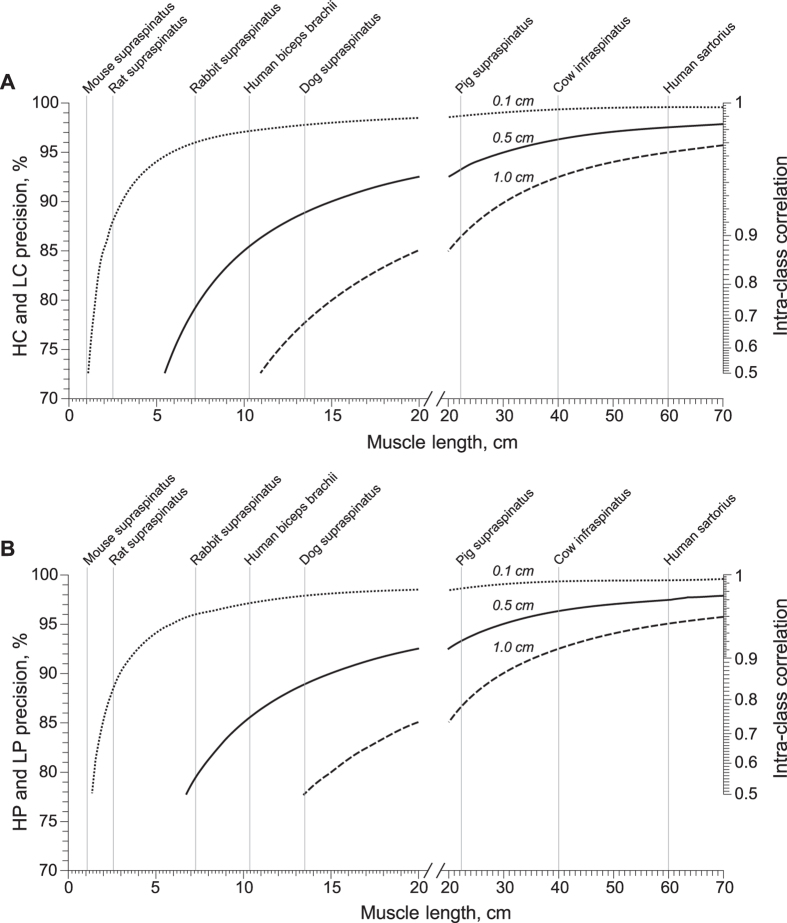
Electrical impedance myography (EIM) reference values of electrodes’ precision and intra-class correlation coefficient (ICC) for different muscle lengths. We considered three realistic cases of electrode positioning, from more to less precise 0.1 cm (dotted), 0.5 cm (solid), 1 cm (dashed), affecting the current (**A**) and voltage (**B**) electrodes separately and starting with an ICC at least 0.5. The minimum muscle lengths measurable are, respectively, {1, 5.4, 10.8} cm with 72% the minimum precision on the current electrodes position (**A**), and {1.2, 6.8, 13.2} cm with 78% the minimum precision on the voltage electrodes (**B**). Muscles shown: human sartorius^70^; mouse supraspinatus, rat supraspinatus, and rabbit supraspinatus^71^; human biceps brachii^72^; dog supraspinatus, pig supraspinatus, and cow infraspinatus^73^. Additional architectural properties of muscles in other vertebrate species can be found elsewhere^73^,^74^,^75^. Data with an ICC below 0.5 are not shown. Electrode nomenclature: HC, high current; HP, high potential; LP, low potential; and LC, low current.

Normative values of the electrode precision required for measuring muscle across different animal species are shown in Fig. 6. In the figure, we analyzed three realistic scenarios of positioning errors affecting the current (Fig. 6A) and voltage (Fig. 6B) electrodes separately, from more to less precise, {0.1, 0.5, 1} cm, starting with an ICC at least 0.5. One can see in Fig. 6 that the minimum length of muscle that can be measured is, respectively, {1, 5.4, 10.8} cm with 72% the minimum precision on the current electrodes position (A), and {1.2, 6.8, 13.2} cm with 78% the minimum precision on the voltage electrodes (B). As the precision of positioning the electrodes increases, both the ICC coefficient and the minimum length of muscle that can be measured increase. The minimum width of the muscle is {0.2, 1.0, 2.1} cm with a minimum precision above 50% in all electrodes. From a practical view, one can use data in Fig. 6 as a reference guide to determine, given a muscle length, the minimum electrode precision necessary and the expected EIM repeatability.

## Discussion

Common use of standard electrophysiological tests for recording muscle potentials in neuromuscular diagnosis requires the accurate placement of electrodes^35^. The effect that electrode placement has on the patterns of responses measured by CMAP^3^,^4^,^5^,^7^,^36^,^37^,^38^,^39^,^40^ and electromyography (EMG)^41^,^42^,^43^,^44^,^45^,^46^ requires *ad hoc* adjustments to maximize data reliability and validity. In this paper, we showed that the reliability of EIM as a biomarker for the assessment of muscle is also affected by the precise positioning of the electrodes.

We conclude the reliability of EIM depends on electrode interspacing and the positioning in each direction. In fact, we found the influence on EIM’s resistance and reactance values caused by a poor placement of the electrodes is the same. These effects turned out to be independent from the electrical properties of muscle; therefore they can be applied equally in healthy and diseased muscles. Moreover, with EIM, the effect of electrode positioning in the amplitude of muscle resistance and reactance data is the same regardless of the muscle measured. This is in great contrast to CMAP, for example, where the muscle area of maximum CMAP amplitude depends on the specific muscle being studied^4^. Another major difference with respect to CMAP or EMG is that, with the novel method presented, one can predict and correct the increase or decrease effects seen in the resistance and reactance data caused by an electrode misplacement without having to resort to the trial-and-error testing required for example in CMAP recording technique.

Importantly, changing the frequency measured does not improve EIM’s reproducibility with electrode positioning errors. This result contradicts the observation made in ref. 29 (see Fig. 2 therein), where the author suggested that bringing the current electrodes close to the potential electrodes had the effect of increasing the reactance at frequencies only above 500 kHz. Our results reveal that bringing the current electrodes close to the potential electrodes has the same effect on the resistance and reactance in a frequency independent manner (Table 3).

Further, our analytical framework also allows the possibility of making quantitative estimates of the precision needed for positioning the electrodes to achieve a pre-specified EIM reproducibility (Fig. 6). This result is particularly important when attempting to measure small muscles, e.g. in newborns or rodents, where ensuring a small electrode placement uncertainty may be challenging using gel-adhesive electrodes, especially if there is no reference coordinate, e.g. a marker in the skin^47^. In these situations, a metal electrode array as the ones used in^18^,^48^,^49^,^50^ can be an alternative. These metal electrode arrays have an interelectrode distance that is fixed and can be machined so that variations in the electrode gaps is on the order of hundreds of microns. While using a metal electrode array will contribute to a reduction in data variability caused by a non-existent misplacement of the electrodes, the use of a metal electrode array does not necessarily mean the EIM’s reproducibility will be higher. Metal electrodes have greater skin-electrode contact impedance than gel-adhesive electrodes and that may adversely affect the EIM data at frequencies in the kHz range^51^,^52^,^53^,^54^. A good practice to mitigate skin-electrode contact impedance consists of cleaning the metal electrodes with alcohol and moistening the skin with saline before measurements. Recent advances in micro and nanotechnologies offer alternatives to wet and metal electrodes for measuring EIM. Li *et al*. recently proved the reproducibility (ICC > 0.9) of microneedle electrode arrays (MEAs) measuring EIM in patients with neurogenic myopathy^55^. These MEAs can penetrate (<200 *μ*m) the stratum corneum painlessly thus reducing the influence of skin layer and electrode-skin contact impedance affecting conventional wet and metal electrodes. Like metal strip electrode arrays, MEAs are reusable. Also, the size of a metal electrode array can be inconvenient when measuring large muscles, for example in horses^56^,^57^.

In human studies, however, the type of electrode needed will also depend on other clinical considerations beyond the electrode array’s size. For example, in the clinical study (clinicaltrials.gov) NCT01491555, we used a handheld probe with metal electrodes to minimize discomfort on children with Duchenne muscular dystrophy and reduce the time required to collect data^58^. In trial NCT02118805, using adhesive electrodes is not an option for measuring the tongue (i.e. genioglossus muscle) in patients with bulbar dysfunction^59^. Instead, we developed a custom-made prototyped tongue array using a tongue depressor and configured with four stainless steel electrodes and wires to interface with the impedance device. In contrast, ongoing studies evaluating the facial muscles, makes such metal electrodes very difficult to employ given poor skin contact relating to oils and non-uniformities in the skin, bony prominences, and facial hair. In this case, employment of gel adhesive electrodes is an alternative. Other studies have measured EIM using gel-adhesive electrodes to detect injury in the lower limb of professional football players^60^,^61^.

Among the limitations of this work, we assumed the electrodes were in direct contact with muscle to make the analysis feasible, an assumption that is satisfied *in vitro*^62^,^63^ and *in vivo*^49^,^64^,^65^,^66^,^67^,^68^. A more accurate impedance analysis should consider the anisotropic properties of the skin, subcutaneous fat and muscle layers^69^. For the same reason as above, we considered the electrodes as point sources and yet the electrodes are not dimensionless. In our analysis, the assumption of muscle being larger than the size of the electrode array reflects this reality well. Finally, we only considered electrodes’ misplacements in the longitudinal and transverse directions separately rather than a more general case where electrode positioning artifacts could occur simultaneously in the two or three directions at once. Despite all these limitations, the discrepancy found between the experimental and theoretical data is below 10% (Table 3), which lends more weight to the validity of the analytical approach presented.

In summary, our goal with this paper was to study the influence of electrode positioning on EIM and to create normative data for the use of this technique in the evaluation of muscle across animal species. By providing a detailed analysis on the effect of electrode placement in EIM, we hope that standardization and objective comparability of future clinical and pre-clinical studies will be enhanced.

## Methods

### Finite element model

Finite element model simulations were done in the frequency domain using the AC/DC Module, Electric Currents Physics in Comsol Multiphysics software (Comsol, Inc, 5.0, Burlington, MA) to validate the theoretical results. A semi-infinite medium was approximated in Comsol by creating a cube with dimension *h* = 100 m, 2000 times the dimension *h*_*e*_ = 0.05 m of the copper electrodes. The cube had relative permittivity *ε*_*r*_ = 1 · 10^4^ (dimensionless) and real conductivity σ = 0.1 Sm^−1^ at 50 kHz, the frequency at which the simulations were conducted. The tetrapolar electrode array was placed on the positive *z* face of the cube with the electrodes spaced along a line parallel to the *x*-axis with a distance *d* = 1 m between the midpoint of each electrode. The midpoint of the array was located at *x* = *h*/2 and *y* = *h*/2. The dimensions of m facilitate the meshing of the FEM simulations and do no affect Δ*R*/*R* and Δ*X*/*X*, the latter depends on the % of distance variation between the electrode positioning with misplacements and the uniform electrode spacing *d*.

The four copper electrodes were modeled as cubes with sides of length *h*_*e*_, relative permittivity *ε*_*r*_ = 1 (dimensionless) and real conductivity *σ* = 5.998 · 10^7 ^Sm^−1^ (values specified at the same frequency). The electrode nearest the coordinate reference point (*x* = *y* = 0) was the high current (source) electrode with *I* = 1 A, chosen so the voltage was directly proportional to the impedance. Moving along the positive *x*-axis, the next two electrodes were the high and low potential voltage electrodes, respectively. The fourth electrode was the low current (sink) electrode, modeled as a ground. The component of the electric current normal to the surface was defined to be null for non electrode-cube boundaries, so current did not flow out of the model into nothingness. The model was meshed in Comsol using tetrahedral elements, resulting in a mesh having 107,213 elements and an approximately 8 s solution time (16 GB RAM, Intel Core i7-3770 CPU @ 3.40 GHz).

Variations of the FEM model were run using Comsol LiveLink toolbox via MATLAB (Mathworks, 2015a, Natick, MA). The single electrode misplacements *dx* and *dy* were parameters in the simulations in *x* and *y* axes, respectively. Single electrode positioning errors were assumed to be smaller than the uniform electrode distance *d* also considering the electrode size. This condition prevents the electrodes misplaced from touching with each other. This condition also ensures the convention for the electrodes arrangement will remain the same: the current electrodes are the outer electrodes, and the high potential electrode is the closest to the high current electrode. Thus, we restricted ourselves *dx* and *dy* ranging from −0.75 m to 0.75 m by increments of 0.25 m, this *dx*/*d* and *dy*/*d* up to ±75% with increments of 25%. For the same reasons, double electrode positioning errors were assumed to be smaller than half the uniform electrode distance and the electrode size, i.e. *dx*_1,2_ and *dy*_1,2_ between ±47.5% with increments of 5%.

### Experimental protocol

The quadriceps electrical impedance was measured with SFB7 (Impedimed Ltd., Queensland, Australia) using gel-adhesive electrodes (70010-K/C/12, Ambu, Denmark) in the non-dominant thigh of *n* = 5 healthy subjects (mean 27.4 years, std 6.5 years). The electrode wires were cut at 5 cm and connected by cable clamps to instrument cables. The subjects had been in a supine position for at least 4 minutes with the arms abducted from the body 15 degrees, and the legs comfortably separated. Electrodes sites were cleaned with alcohol prior to affixing the electrodes and the equipment cables were not intertwined, touching the ground, metal objects, nor near other voltage equipment. At the beginning of the experiment, we attached all the electrodes required so they could be reused to avoid irritating the skin by repositioning new electrodes. We ensured that each electrode did not touch the neighboring electrodes to prevent introducing errors due to a different electrode contact area. We assumed that the presence of the additional electrodes had no effect on the impedance measured.

The more distal electrode was used as reference and placed 5 cm from the patella. The remaining electrodes were placed contiguously longitudinally (*x*-axis) and transversely (*y*-axis) relative to the long axis of the rectus femoris (shown in Fig. 2). The convention adopted for misplacing the electrodes was as follows: we moved the electrodes distally and proximally when *dx* was positive and negative, respectively. The electrodes were moved laterally and medially when *dy* was positive and negative, respectively. The uniform electrode spacing was *d* = 7 cm. The distance between centers of additional electrodes was *dx* = *dy* = 1.5 cm, giving {−75, −50, −25, 25, 50, 75}% the electrodes’ positioning errors. To keep the number of measurements reasonably small, we only measured the impedance of the quadriceps with single electrode positioning errors in each axis separately. In all, each volunteer was measured 50 times and the experiment time was 15 min approximately. During the experiment, the subject remained in the same position to avoid data fluctuations. The study was approved by the institutional review board for the Beth Israel Deaconess Medical Center, and written informed consent was obtained from all participants. All procedures were conducted according to the Declaration of Helsinki.

### Data analysis

We calculated the resistance and reactance relative errors in MATLAB from simulated and experimental data using *e*_real_ in equation 2. To validate the framework, we quantified the percentage difference between the mean experimental resistance and reactance relative errors and the predicted relative errors using Table 2 in each axis separately. The case with uniform electrodes’ distance was not considered in the analysis because, by definition, resistance and reactance data did not have errors. Mann-Whitney test (two-tailed) was used for single- and multi-frequency data comparisons in Table 3. The statistical significance was set at *p* < 0.05.

## Additional Information

**How to cite this article**: Sanchez, B. *et al*. Guidelines to electrode positioning for human and animal electrical impedance myography research. *Sci. Rep*. **6**, 32615; doi: 10.1038/srep32615 (2016).

## Supplementary Material

Supplementary Information

## Figures and Tables

**Figure 1:**
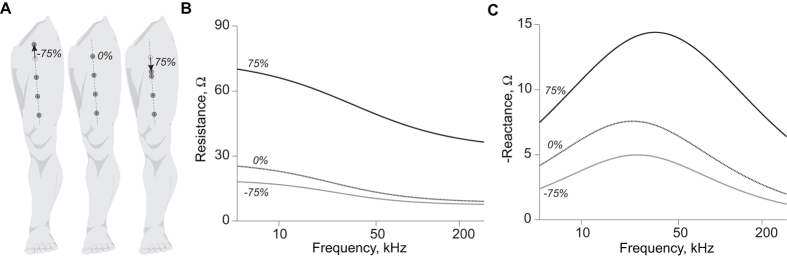
Example of the effect of electrode misplacement in electrical impedance myography. (**A**) Schematic illustrating the misplacement of the high current (HC) electrode in Figure 2 proximally towards the hip joint (−75%) and distally towards to the high potential (HP) electrode (75%). Effect on the resistance *R* (**B**) and reactance *X* (**C**). The reference resistance and reactance data measured with an evenly spaced electrode array (0%) are shown with dotted lines for comparison purposes. The consequence of a misplacement of the current source electrode is an increase or decrease in the resistance and reactance data.

**Table 1:** Published studies evaluating the effects of electrode(s) positioning using electrical impedance.

	Electrode(s)	Misplacement		Impedance result(s)	Frequency (kHz)	Normalized to *d*	Validation method	Application
		*x*-axis	*y*-axis					
Dunbar *et al*.^22^	HC, HP, LC, LP, HC&HP&LC&LP	const.	const.	unk.	50	No	Experiment	BIA
Lozano *et al*.^20^	HC, HC&HP&LC&LP	unk.	unk.	Δ|*Z*|/|*Z*|	8, 125	No	Experiment	BIA
Cornish *et al*.^23^	HC&HP&LC&LP	unk.	unk.	Δ*R*/*R*	5, 50, 100	No	Experiment	BIA
Rutkove *et al*.^28^	HC&LC, HP&LP	const.	const.	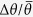	50	No	Experiment	EIM
Stahn *et al*.^76^	HP&LP	const.	unk.	*R*	50–500	No	Theory, experiment	BIA
Moon *et al*.^24^	HC&HP, LP&LC	const.	unk.	*R*_0_, *R*_∞_, *R*_i_	3–1000	No	Experiment	BIA
Shiffman^29^	HP&LP	const.	unk.	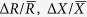	2–2000	No	Experiment	EIM
Grisbrook *et al*.^25^	HC&HP&LC&LP	unk.	unk.	*R*_0_, *R*_∞_, *R*_i_	3–1000	No	Experiment	BIA
In this work	HC, HP, LC, LP, HC&LC, HP&LP, HC&HP, HC&LP, HP&LC, LP&LC	ind.	ind.	Δ*R*/*R*, Δ*X*/*X*, Δ*θ*/*θ* = 0	3–1000	Yes	Theory, simulation, experiment	EIM

Δ|*Z*|/|*Z*|, relative change magnitude; Δ*R*/*R*, relative change resistance; 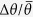, relative change spatially averaged phase; *R*, resistance; *R*_0_, modeled resistance at zero frequency; *R*_∞_, modeled resistance at infinite frequency; *R*_i_, intracellular resistance calculated from *R*_0_ and *R*_∞_; 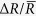 & 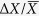, relative change spatially averaged resistance and reactance, respectively; Δ*X*/*X*, relative change reactance; Δ*θ*/*θ*, relative change phase; *d*, distance between electrodes. Abbreviations: EIM, electrical impedance myography; BIA, bioelectrical impedance analysis; const., constant; ind., independent; unk., unknown; HC, high current electrode; HP, high potential electrode; LP, low potential electrode; LC, low current electrode.

**Table 2:** Predicted relative errors due to single and double electrode misplacements.

Electrode(s)	Δ*e*/*e*
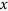 -axis	
HC	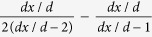
HP	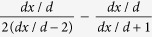
LP	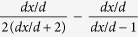
LC	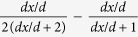
HC&LC	HC + LC
HP&LP	HP + LP
HC&HP	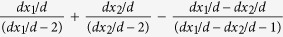
HC&LP	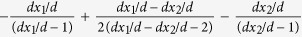
HP&LC	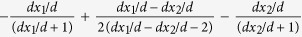
LP&LC	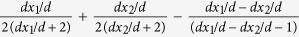
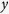 -axis	
HC	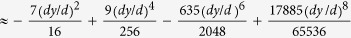
HP	HC
LP	HC
LC	HC
HC&LC	≈HC + LC
HP&LP	HC&LC
HC&HP	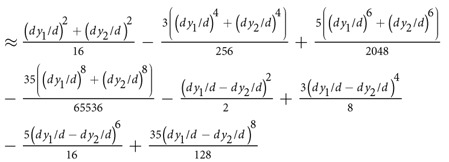
HC&LP	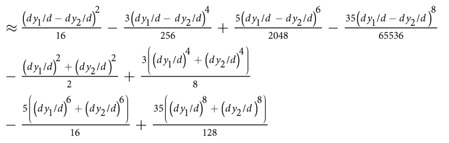
HP&LC	HC&LP
LP&LC	HC&HP

The electrodes misplacements are *dx*_{},1,2_ and *dy*_{},1,2_ whereas *d* is the uniform electrode spacing. The subscript refers to the electrode(s) with positioning error(s). The influence of electrode positioning is studied in longitudinal *x* and transverse *y* directions separately. Electrodes nomenclature: HC, high current; HP, high potential; LP, low potential; LC, low current.

**Table 3:** Percentage difference between the mean measured resistance and reactance relative error (*n* = 5 subjects) and the predicted relative error at 50 kHz and averaged over 133 frequencies between 10 and 200 kHz.

	kHz	%	HC	HP	LP	LC
*x*-axis	50	±25{±25, ±50}	2.4 ± 1.95.9 ± 3.5	3.8 ± 2.39.8 ± 4.3	4.5 ± 3.25.8 ± 3.9	4.2 ± 3.36.2 ± 4.6
	10–200†	{±25, ±50}	6.0 ± 3.5	9.9 ± 4.2	6.0 ± 4.2	6.4 ± 4.3
*y*-axis	50	±25{±25, ±50}	1.6 ± 1.21.9 ± 0.9	3.7 ± 1.54.7 ± 2.5	4.4 ± 2.16.5 ± 3.0	2.4 ± 1.02.6 ± 1.2
	10–200†	{±25, ±50}	2.0 ± 0.6	4.5 ± 2.4	6.3 ± 3.5	2.6 ± 1.3

The electrodes’ misplacements considered in the *x* and *y* axes are {−25, 25}% and {−50, −25, 25, 50}%. The comparison between multi-frequency and single-frequency 50 kHz data was found to be not statistically different (*p* values above 0.55 in both directions). Electrodes nomenclature: HC, high current; HP, high potential; LP, low potential; LC, low current. Values are reported as mean ± standard deviation.

^†^133 frequencies.

**Table 4:** Quantitative estimates of the maximum electrode movement allowed in % positioning the electrodes in the *x* and *y* axes to achieve a pre-specified reproducibility given by the intra-class correlation (ICC).

ICC	*x*-axis, %		*y*-axis, % All
	HC, HP	LP, LC	
≥0.9	13.6	8.7	37.5
≥0.8	18.5	12.9	44.6
≥0.7	22.2	16.4	46.6
≥0.6	24.8	19.6	47.5
≥0.5	26.9	22.7	48.3
≥0.4	30.0	26.1	48.9
≥0.3	33.2	29.2	49.5
≥0.2	36.1	32.7	50.0

The maximum permissible electrode misplacement decreases with increasing the reproducibility. Electrodes nomenclature: HC, high current; HP, high potential; LP, low potential; LC, low current.
