# Supplementary material for: Guidelines to electrode positioning for human and animal electrical impedance myography research
Source: Sci Rep. 2016 Sep 2;6:32615. doi: 10.1038/srep32615 (PMC5009322; doi:10.1038/srep32615)
Supplement: Supplementary Information [file srep32615-s1.pdf]

Supplementary materials for

Guidelines to electrode positioning for human and animal electrical impedance research

Benjamin Sanchez[1], **Adam Pacheck**[1], **and Seward B Rutkove**[1]

[1]Department of Neurology, Division of Neuromuscular Diseases, Beth Israel Deaconess Medical Center, Harvard Medical School, Boston, MA 02215-5491, USA.

E-mail: `bsanchez@bidmc.harvard.edu`

Received 20 April 2016, reviewed 22 June 2016, revised 12 July 2016

Appendix

Here we lay the theoretical foundations necessary to analyze the muscle impedance due to electrode misplacements. Consider the Ohm's law in its vector form,

$$\boldsymbol{J} = \frac{1}{\rho} \cdot \boldsymbol{E}, \tag{1}$$

where $\boldsymbol{J}$ is the current density vector in Am^{-2}, $\boldsymbol{E}$ is the electric field vector in Vm^{-1}, and ρ is the material-dependent complex resistivity in Ωm. Assuming the high current, high potential, low potential, and low current electrodes as point sources, the current density across the a semi-infinite homogenous material equals the current I injected spread across the surface area of a half sphere [1],

$$\boldsymbol{J} = \frac{I}{\frac{1}{2}\left(4\pi r^2\right)} = -\frac{1}{\rho} \cdot \frac{dV}{dr}. \tag{2}$$

Solving the first-order differential (2) by integrating both sides of equation and assuming the complex conductivity is isotropic gives the potential V measured at a distance r from the current electrodes, namely

$$V(r) = \frac{\rho I}{2\pi r}. \tag{3}$$

The voltage measured by the high and low potential electrodes depends, respectively, on the distance between the high potential (HP) electrode and the current electrodes,

$$V_{\mathrm{HP}} = \frac{\rho I}{2\pi}\left(\frac{1}{d} - \frac{1}{2d}\right), \tag{4}$$

and the distance between the low potential (LP) electrode and the current electrodes,

$$V_{\mathrm{LP}} = \frac{\rho I}{2\pi}\left(\frac{1}{2d} - \frac{1}{d}\right), \tag{5}$$

where d is the uniform electrode spacing (Supplementary figure 2A). The "error-free" impedance with uniform electrode spacing follows easily from the superposition of the high and low potentials in (4)-(5), i.e.

$$Z = \frac{V_{\mathrm{HP}} - V_{\mathrm{LP}}}{I} = \frac{\rho}{2\pi} \cdot \frac{1}{d}. \tag{6}$$

The reader can follow the same reasoning to find the expression of the impedance Z_e with single and double electrode(s) error(s) shown in Supplementary figure 1. The errors in Table 2 can be found by rewriting Z_e according to (1) in the paper, using Z defined in (6).

The results in Table 2 can be generalized without fundamental problems to a non-uniform linear electrode array following the aforementioned steps. If the distance between the potential electrodes is D (Supplementary figure 2B), then the "error-free" impedance (6) becomes

$$Z = \frac{\rho}{2\pi} \cdot \frac{2D}{d(d+D)}. \tag{7}$$

Note (6) and (7) are equivalent when $D = d$.

[1] Malmivuo, J. & Plonsey, R. *Bioelectromagnetism: Principles and Applications of Bioelectric and Biomagnetic Fields* (New York: Oxford University Press, 1995).

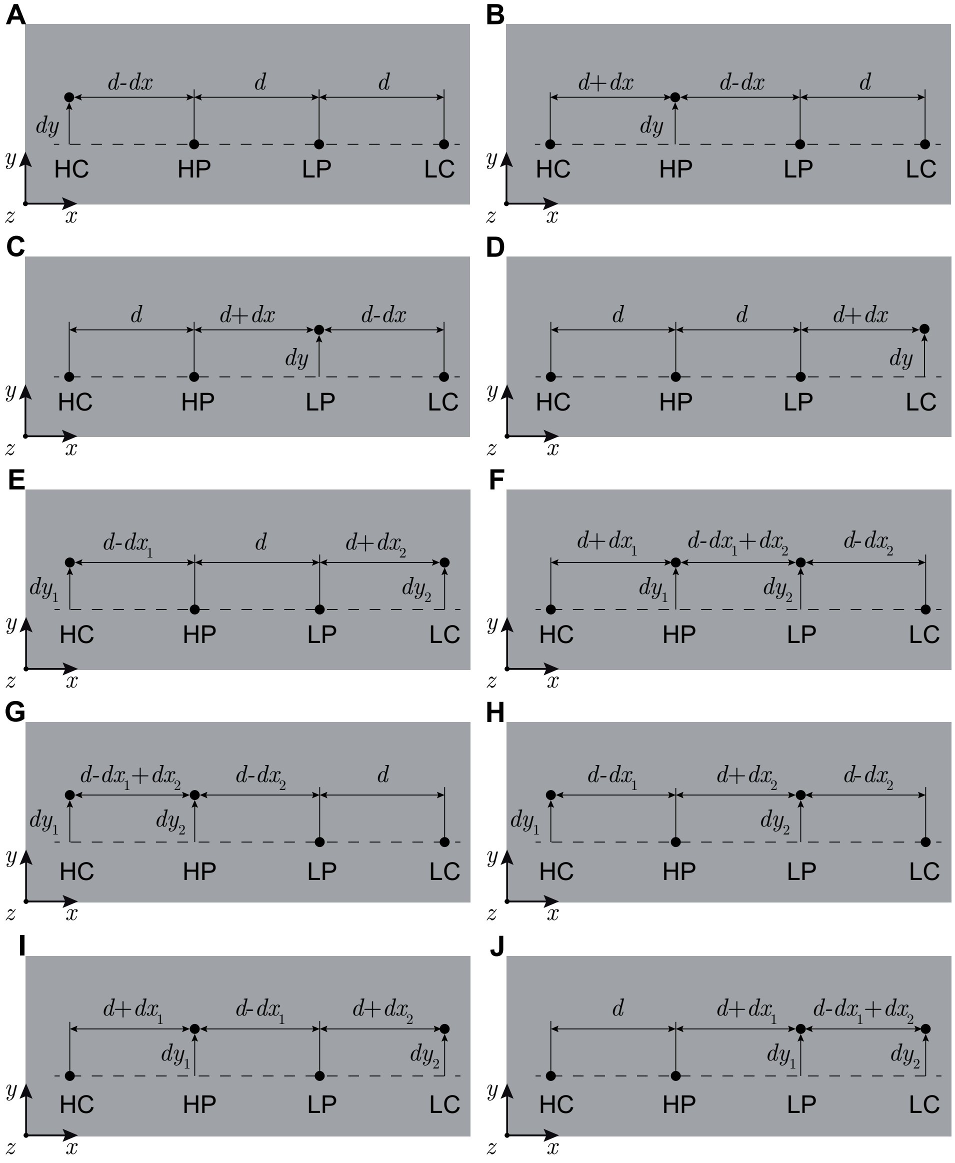

Supplementary Figure 1: Two-dimensional representation of a surface electrical impedance measurement with single (A–D) and double (E–J) electrode misplacements: (A), high current; (B), high potential; (C), low potential; (D), low current; (E), high and low current; (F), high and low potential; (G), high current and high potential; (H), high current and low potential; (I), high potential and low current; and (J), low potential and low current. The point source electrodes (in black) are positioned above the muscle's surface represented as a semi-infinite homogenous volume (in gray). We use as a convention that positive electrode changes in position moves the electrodes towards increasing values in the xy plane. The dotted line shows the x-axis in which the electrodes are aligned when there is no electrode positioning errors in the y-axis. The electrodes' naming convention adopted for the current source and current sink electrodes is, respectively, high current (HC) electrode and low current (LC) electrode. The voltage sensing electrodes are defined as high potential (HP) electrode and low potential (LP) electrodes according to their relative position with respect to the current electrodes.

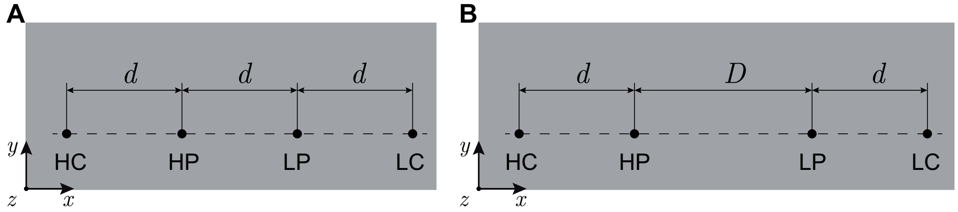

Supplementary Figure 2: Two-dimensional representation of a surface electrical impedance measurement without electrode misplacements considering an evenly (A) and non-evenly (B) spaced electrode array. The point source electrodes are shown in black circles, placed above the muscle surface represented as a semi-infinite homogenous volume (in gray). The dotted line shows the x-axis in which the electrodes are aligned when there is no electrode misplacement in the y-axis. Electrode nomenclature: HC, high current; HP, high potential; LP, low potential; and LC, low current.
